# Sphingosine Kinase 1 and Cancer: A Systematic Review and Meta-Analysis

**DOI:** 10.1371/journal.pone.0090362

**Published:** 2014-02-27

**Authors:** Yun Zhang, Yan Wang, Zhi Wan, Shiping Liu, Yu Cao, Zhi Zeng

**Affiliations:** 1 Department of Emergency, West China Hospital, West China School of Medicine, Sichuan University, Chengdu, China; 2 Department of Epidemiology, School of Public Health, Jilin Medical College, Jilin, China; National Cancer Center, Japan

## Abstract

**Background:**

Sphingosine kinase 1 (SK1) is a key regulator of the dynamic ceramide/sphingosine 1-phosphate rheostat balance and important in the pathological cancer genesis, progression, and metastasis processes. Many studies have demonstrated SK1 overexpressed in various cancers, but no meta-analysis has evaluated the relationship between SK1 and various cancers.

**Methods:**

We retrieved relevant articles from the PubMed, EBSCO, ISI, and OVID databases. A pooled odds ratio (OR) was used to assess the associations between SK1 expression and cancer; hazard ratios (HR) were used for 5-year and overall survival. Review Manager 5.0 was used for the meta-analysis, and publication bias was evaluated with STATA 12.0 (Egger’s test).

**Results:**

Thirty-four eligible studies (n = 4,673 patients) were identified. SK1 positivity and high expression were significantly different between cancer, non-cancer, and benign tissues. SK1 mRNA and protein expression levels were elevated in the cancer tissues, compared with the normal tissues. SK1 positivity rates differed between various cancer types (lowest [27.3%] in estrogen receptor-positive breast cancer and highest [82.2%] in tongue squamous cell carcinoma). SK1 positivity and high expression were associated with 5-year survival; the HR was 1.86 (95% confidence interval [CI], 1.18–2.94) for breast cancer, 1.58 (1.08–2.31) for gastric cancer, and 2.68 (2.10–3.44) for other cancers; the total cancer HR was 2.21 (95% CI, 1.83–2.67; P < 0.00001). The overall survival HRs were 2.09 (95% CI, 1.35–3.22), 1.56 (1.08–2.25), and 2.62 (2.05–3.35) in breast, gastric, and other cancers, respectively. The total effect HR was 2.21 (95% CI, 1.83–2.66; P < 0.00001).

**Conclusions:**

SK1 positivity and high expression were significantly associated with cancer and a shorter 5-year and overall survival. SK1 positivity rates vary tremendously among the cancer types. It is necessary to further explore whether SK1 might be a predictive biomarker of outcomes in cancer patients.

## Introduction

Sphingolipids are structural and functional components of biological membranes [1], and their metabolites, including sphinganine, ceramide, sphingosine, and sphingosine 1-phosphate (S1P), have emerged as critical players in a number of fundamental biological processes. For example, these metabolites act as bioactive mediators in various cellular processes, including survival, proliferation, differentiation, and apoptosis [2], [3]. Moreover, sphingolipids are known to be involved in almost every type of disease [4]. They are reported to have regulation effect n cancer pathogenesis, progression, angiogenesis, proliferation, migration, inflammation, drug resistance, and cell death (apoptosis, necrosis, autophagy, and anoikis) [5], [6]. Sphingosine kinases (SphKs), including sphingosine kinase type 1 (SK1/SphK1) and sphingosine kinase type 2 (SK2/SphK2), catalyze the phosphorylation of sphingosine to S1P and are crucial regulators of the balance among ceramides, sphingosine, and S1P [7]. Unlike SK2, SK1 does not contain any transmembrane domains. In addition, their tissue distributions differ in human beings, as well as in animals [8]–[10], and their opposing functions in sphingolipid metabolism have also been reported [11]. Specifically, SK1 has become a cancer research hotspot in cancer and has recently been considered a bona fide oncogene [12].

Much evidence has shown that SK1 can be detected in tumor tissues; notably, SK1 was reported to be overexpressed in most studies [13]–[16]. Immunohistochemistry (IHC), polymerase chain reaction (PCR), and western blotting (WB) are usually used in such studies. The data showed that relative to paired non-cancer tissues, elevated SK1 expression ranges from 2- to 8-fold at both the mRNA and protein levels in some cancers, including breast, lung, ovarian, stomach, and colon cancers. Meanwhile, some studies found that SK1 overexpression might be related to cancer metastasis, reduced survival time, and poor prognosis [17], [18]. Therefore, SK1 was suggested as a novel biomarker of clinical prognosis in some cancers [19]. However, no systematic reviews or meta-analyses have discussed the role and clinical significance of SK1 in cancer. Therefore, we performed a meta-analysis to investigate the relationship between SK1 expression and various cancers and the possibility that SK1 might be used as a cancer biomarker.

## Materials and Methods

### Search strategy and study selection

Electronic databases, including the English PubMed, EBSCO, ISI, OVID, ACS, and Cochrane Library databases and the Chinese VIP, Wan Fang, and China National Knowledge Infrastructure (CNKI) databases, and Google Scholar were searched for publications from inception to September 2013. The search keywords and terms were “sphingosine kinase type 1” OR “sphingosine kinase 1” OR “Sphk1” OR “Sphk-1” OR “SK1” OR “SK-1” OR “SK-I” AND “cancer” OR “tumor” OR “neoplasm” OR “carcinoma.” Only Chinese- and English-language papers were included. All articles were imported into EndNote X6 software (Thomson Reuters Corporation, New York, NY, USA) to eliminate duplicates.

All titles and abstracts were read by 2 independent reviewers (Yun ZHANG and Yan WANG) to select eligible studies. Next, the full texts were independently read and carefully checked to exclude ineligible studies. Disagreements were resolved via consensus.

### Study selection criteria

The inclusion criteria for primary studies were as follows: (1) human studies; (2) cancer types and detection method were clearly described; and (3) SK1 expression was reported.

The exclusion criteria for primary studies can be summarized as follows: (1) reviews, abstracts, and case reports; (2) animal or cell studies; (3) no description of detection methods; (4) a total sample size < 10; (5) a lack of SK1 expression data or the IHC, PCR, and WB results were only reported in figures; (6) the hazard ratios (HR) and 95% confidence intervals (CI) of survival analyses were not reported or could not be calculated from other data; and all articles with the data from the same patient population, except for the most recent report.

### Data extraction

A data collection form was designed before extracting data. The final included articles were assessed independently by 2 reviewers (Yun ZHANG and Yan WANG). The extracted data included the first author, year of publication, and country; cancer types, specimen types, and detection methods; the number of patients in the experimental and control groups; the number of cancer, adjacent non-cancer, and benign samples and the number of SK1-positive (high expression or a score ≥ ++) and negative patients (low expression or a score of – or +); the mRNA/protein expression levels in cancer and non-cancer tissues; the 5-year survival, overall survival (OS), disease-specific survival (DSS), and disease-free survival (DFS) outcomes and the follow-up duration. Among these, if the HR was not originally reported, we calculated or extracted this data according to the methods described by Tierney (2007) or Parmar (1998) [20], [21].

### Statistical methods

The Review Manager software (version 5.02 for Windows; The Cochrane Collaboration, 2009) was used for the meta-analysis, and STATA 12.0 software (StataCorp, College Station, TX, USA) was used to analyze the publication bias (Egger’s test). According to the Review Manager Handbook, for dichotomous data such as the number of patients with SK1 positivity/high expression in cancer and non-cancer tissues, the odds ratios (OR) and 95% CI were combined to provide effective values. For continuous data such as SK1 activity, the standardized mean difference (SMD) and 95% CI were used to estimate effective values. For the association between SK1 and OS, 5-year survival was described as a HR and 95% CI. Statistical heterogeneity was examined with the Cochrane Q-test (significant at P < 0.1) and *I* ^2^ value. When P < 0.1 or *I* ^2^ < 50%, a fixed-effect model was used; otherwise, a random-effect model was used. If necessary, a sensitive analysis was also performed to evaluate the influences of individual studies on the final effect. When some studies were omitted or subgroup analyses were adopted and if no decreases in heterogeneity were observed, the qualitative systematic review method was used to describe the results. All P values were 2-sided, and P < 0.05 was considered significant.

## Results

### Search results and characteristics

**Figure 1:**
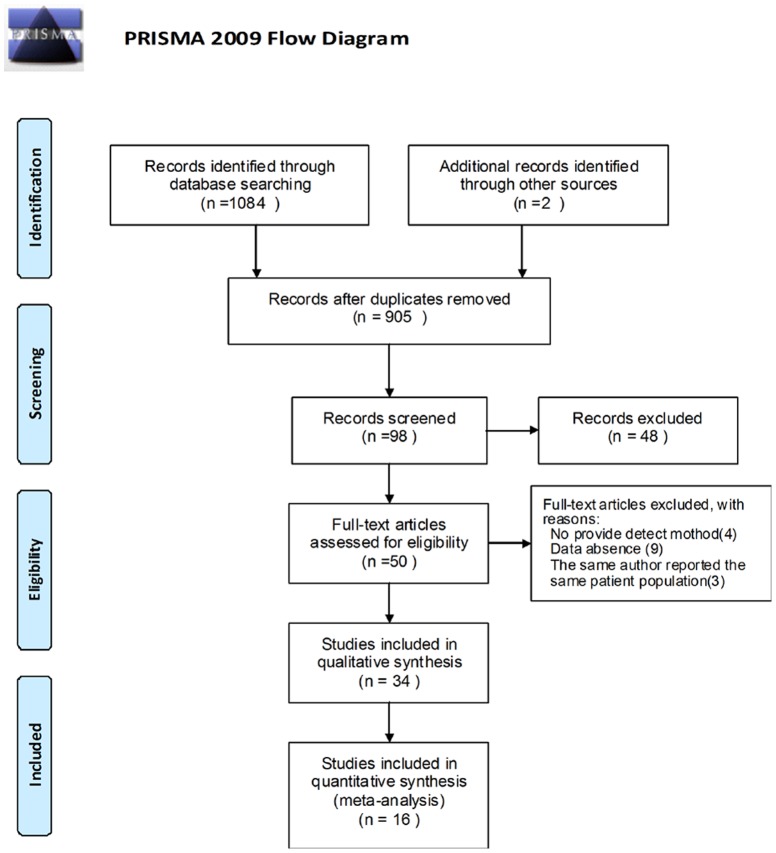
Flow chart of identified eligible studies.

According to the inclusion criteria, 34 studies, including 11 Chinese articles and 23 English articles, were eligible for the final meta-analysis (figure 1) [14]–[16], [22]–[53]. The studies were published between 2005 and 2013. This analysis included 4,673 patients, 19 types of cancer from 7 countries, and 5 types of detection methods. The follow-up duration ranged from 30 months to 12 years. Seven studies were about breast cancer; 6 studies, colon cancer; and 4 studies, gastric cancer. The characteristics of the included studies are shown in Table 1.

**Table 1:** Main characteristics and results of the eligible studies.

First author, year	Country	Ethnicity	Location of Cancer	Stage	Samples	No. pts(e/c)	Detection method	SK1 exp	Cut off	Follow up	HR estimation
Eugen R. 2008	Germany	Caucasian	Breast	Hg I–III	microarray	171/968	IHC	Pr	AP>843	60 m	5S 1.59(1.11–2.28)
Eugen R.2013	Germany	Caucasian	Breast	Cs I–III	TMA	112	IHC	Pr	IRS≥6	120 m	5S 1.29(0.19–8.81) OS 1.24(0.24–6.33)
Racheli E.R. 2010	UK	Caucasian	Breast	SBR I–III	RNA	20(20/20)	PCR	mRNA	NA	NA	NA
Zhang Y. 2010	China	Asian	Breast	Cs I–III	FFPE	77	IHC	Pr	IRS≥4	NA	NA
Jan O. 2012	UK	Caucasian	Breast (ER-)	SBR I–III	TMA	140	IHC	Pr	Hs≥50%	140 m	OS 8.84(4.51–17.33)
Jan O.2013	UK	Caucasian	Breast (ER+)	Cs I–III	FFPE	304	IHC	Pr	Hs≥50%	120 m	5S 1.84(0.97–3.49) OS 2.17(1.38–3.39)
Carol W. 2010	UK	Caucasian	Breast (ER+)	SBR I–III	TMA	304	IHC	Pr	Hs≥2	144 m	5S 1.98(1.0–3.92) OS 1.80(1.10–2.93)
Liu S.Q. 2012	China	Asian	Colon	Ds A-D	Fre/ FFPE	66(66/66)	IHC/WB	Pr	Ws≥2	NA	NA
Su Y.j.2012	China	Asian	Colon	Ds A-D	Fre/ FFPE	66	IHC/PCR/WB	mRNA/Pr	Ws≥2+	NA	NA
Toshihiko K. 2009	USA	Caucasian	Colon	NA	TMA	94	IHC	Pr	Hs≥25%	NA	NA
Jin H. 2010	China	Asian	Colorectal	NA	Fre	48(24/24)	PCR/WB	mRNA/Pr	NA	NA	NA
Roberta R. 2013	Italy	Caucasian	Colorectal	NA	FFPE	50	IHC/WB	Pr	Hs≥50%	NA	NA
Paweł K. 2010	Poland	Caucasian	Endometrial	NA	Fre/ blood	41(23/18)	Ra	Activity	NA	NA	NA
Jian Pan 2011	China	Asian	Esophageal	T0-T3	TMA/Fre/FFPE	124/30pair/12pair	TMA/IHC/WB	Pr	Hs≥25%	96 m	5S 2.60(1.93–3.51) OS 2.62(1.93–3.55)
Liu Y. 2012	China	Asian	Esophageal	T0-T3	Fre	56	PCR/WB	mRNA/Pr	NA	NA	NA
Shang H.X. 2012	China	Asian	Gastric	Cs I-IV	Fre/FFPE	31(31/31)	PCR/IHC	mRNA	IRS≥6	NA	NA
Wen L.2009	China	Asian	Gastric	Cs I-IV	Fre/FFPE	185(175/10)	IHC/PCR/WB	mRNA/Pr	IRS≥6	140 m	5S 6.32(3.80–10.53)
Zhuge Y.H. 2011	China	Asian	Gastric	Cs I-IV	Fre	246(206/40)	IHC	Pr	IRS≥4	60 m	5S 2.06(0.99–4.29) OS 2.06(0.99–4.28)
Zhang L.J. 2013	China	Asian	Gastric	NA	Fre	5pair	IHC/WB	Pr	NA	NA	NA
Guan H.Y. 2009	China	Asian	Glioma	Cs I-IV	FFPE/Fre	243/4	IHC/PCR	Pr	IRS≥8	60 m	5S 2.95(1.46–5.98) OS 2.95(1.46–5.98)
Bao M.Y. 2011	China	Asian	Liver	NA	Fre	100(50/50)	PCR	mRNA	NA	NA	NA
Brianna N.H.2013	USA	Caucasian	HNSCC	NA	Fre	18	IHC/PCR/WB	mRNA/Pr	NA	30 m	OS 0.79(0.14–4.56)
Keisuke S.2011	USA	Caucasian	HNSCC	Cs I–IV	TMA	95(78/17)	IHC	Protein	IRS≥1.5	NA	NA
Uttam K.S. 2010	USA	Caucasian	HNSCC	Cs I–IV	Fre/FFPE	34	IHC/PCR/WB	mRNA/Pr	NA	NA	NA
Satish K.2012	USA	Caucasian	MPM	NA	TMA	75(62/13)	IHC+TMA	Pr	NA	NA	NA
Xu Z. 2010	China	Asian	Neuroblastom	INSS I–IV	FFPE	142	IHC	Pr	NA	NA	NA
Michael G.B.2008	USA	Caucasian	NHL	Cs I–IV	Fre	69(44/25)	IHC/PCR/WB	mRNA/Pr	MGB≥2	NA	NA
Korey R. J. 2005	USA	Caucasian	NSCLC/Breast/Ov/Ut/Rect/SI	NA	FFPE/cDNA	20/40/14/40/16/2	IHC/cDNA probe	mRNA	NA	NA	NA
Bernard M. 2010	France	Caucasian	prostate	Ps 2b–3b	Fre/TMA	30/88	Ra/IHC	Activity/Pr	NA	NA	NA
Nunes J. 2012	UK	Caucasian	Prostate	NA	RBC	108(88/20)	Ra	Activity	NA	NA	NA
Leyre B. 2012	France	Caucasian	prostate	NA	-	139/13pair	IHC/ Ra	Activity	IRS≥3	NA	NA
Liu G.L. 2010	China	Asian	SGC	Cs I–IV	Fre/FFPE	161(159/2)	IHC/PCR/WB	mRNA/Pr	Ws≥6	120 m	5S 2.89(1.56–5.34) OS 2.02(0.92–4.43)
Guan H.Y. 2011	China	Asian	Thyroid	Cs I–IV	FFPE	58(42/16)	IHC	Pr	NA	NA	NA
Yue Z.W. 2012	China	Asian	TSCC	Cs I–IV	FFPE	126	IHC	Pr	Ws≥2	NA	NA

**Abbreviations** FFPE: formalin fixed paraffin embedded; TMA: tissue microarrays; ICH: Immunohistochemistry; PCR: polymerase chain reaction; WB: western-blot; Ra: radioassay; Pr: protein; HNSCC: Head and neck squamous cell carcinoma; TSCC: Tongue squamous cell carcinoma; MPM: Malignant pleural mesothelioma; NHL: non-Hodgkin lymphomas; SGC: salivary gland carcinoma; Ov: Ovary; Ut: Uterus; Rect: Rectum; SI: Small intestine; OS: Overall survival; 5S: Five-year survival rate; Cs: Clinical stage; Hg: Histological grade; SBR: Scarff-Bloom-Richardson; Ds: Dukes' stage; INSS: International Neuroblastoma Staging System; Ps: Pathological stage; IRS; Immunoreactivity scores; AP: Affymetrix Probeset; Hs: Histoscore method; Ws: Weighted score; MGB: hematopathologist; NA: not available.

### SK1 expression in cancer and non-cancer tissues

**Figure 2:**
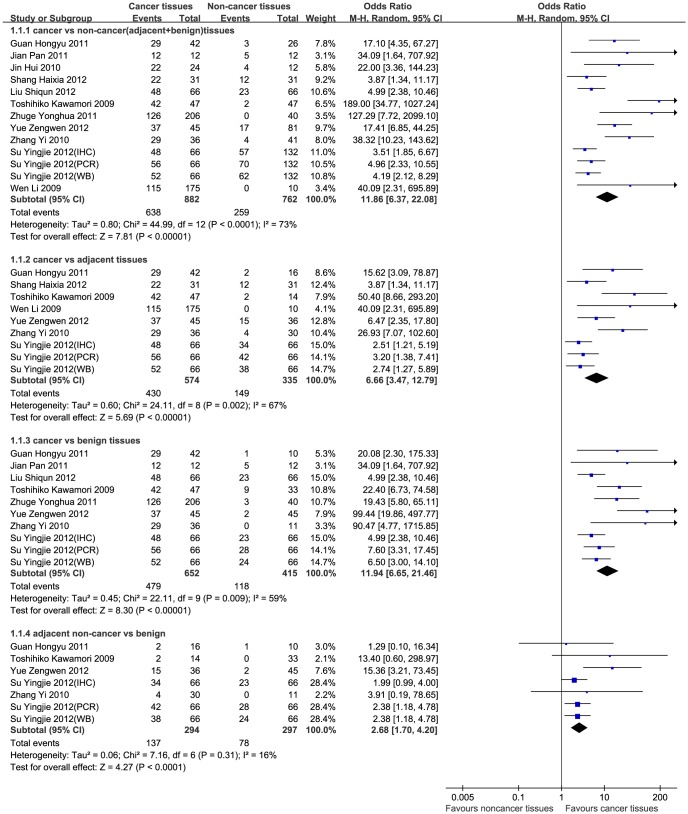
Forest plot of SK1 positivity and high expression in cancer and on-cancer tissues. A subgroup analysis and random-effect model were used. The squares and horizontal lines correspond to the study-specific OR and 95% CI. The diamond represents the summary OR and 95% CI.

Initially, we assessed the association between SK1 positivity/high expression and cancer. Thirteen studies in which SK1 expression was detected by IHC were included in this meta-analysis. Statistical heterogeneities were found in the subgroup analyses, except for that of adjacent non-cancer tissues versus benign tissues; therefore, a random-effect model was applied. The results demonstrated that significant differences in SK1 positivity/high expression rates were found between cancer and non-cancer tissues, cancer and adjacent non-cancer tissues, cancer and benign tissues, and adjacent non-cancer and benign tissues (all P < 0.0001); the respective OR (95% CI) were 11.86 (6.37–22.08), 6.66 (3.47–12.79), 11.94 (6.65–21.46), and 2.68 (1.70–4.20). SK1 positivity/high expression is considered to associate with cancer (Figure 2). To prove the robust results regarding this association between SK1 expression and cancer, a sensitivity analysis was conducted by omitting some of the obviously different studies until an acceptable heterogeneity level was reached (Table 2). In the absence of significant heterogeneity (P > 0.1, *I* ^2^ < 50%) among the studies, a fixed model was adopted and the association was still observed. Therefore, we think that our results were stable and credible.

**Table 2:** Sensitivity analysis of high heterogeneity outcomes in meta-analysis.

		Experimental		control		heterogeneity			Outcomes	
Heterogeneity outcomes	Omitted studies	n	N	n	N	I^2^	*P*	Meta-analysis model	OR(95%CI)	*P*
cancer vs non-cancer	[15], [32], [38], [46]	404	548	236	553	26%	0.21	Fixed	5.32 (3.91, 7.24)	<0.0001
cancer vs adjacent	[40], [45]	252	345	23	106	30%	0.22	Fixed	15.96 (8.42, 30.26)	<0.00001
cancer vs benign	[45], [49]	275	388	20	150	0%	0.62	Fixed	30.15 (15.23, 59.70)	<0.00001

### SK1 mRNA and protein expression level changes in cancers

**Figure 3:**
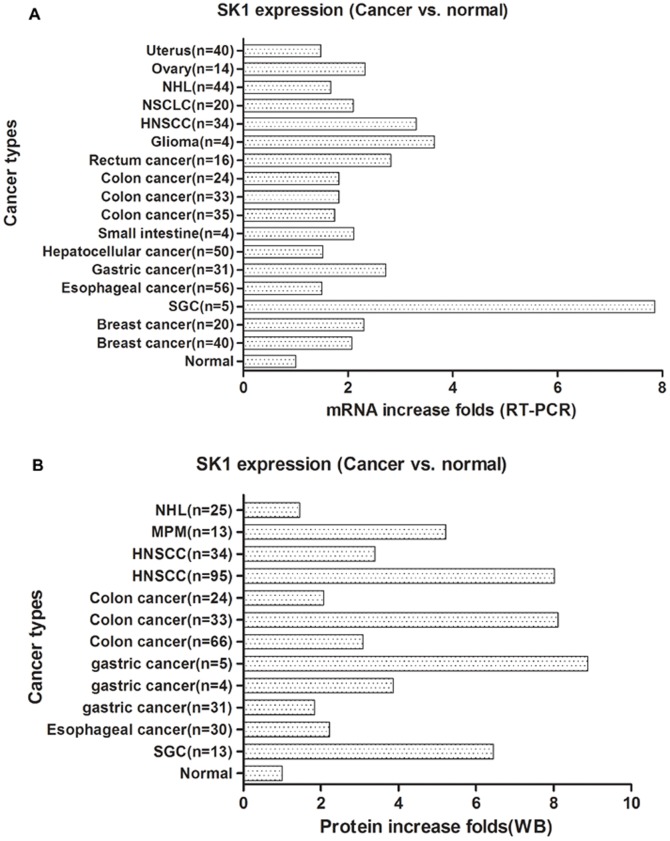
SK1 mRNA and protein-level expression in cancers, compared with normal tissues, via RT-PCR (A) and western blot (B). SK1 expression levels in all normal tissues are assumed to be 1. Histograms symbolize the relative levels of SK1 protein or mRNA expression in different cancers.

Seventeen studies reported SK1 mRNA expression levels, and 12 studies reported SK1 protein expression levels. The sample sizes in these studies ranged from 4 to 95 cases. All outcomes indicated an increasing trend of SK1 mRNA and protein expression levels in cancer tissues. When the SK1 expression levels in normal tissues were assumed to be 1, the mRNA level increases ranged from 1.48-fold in uterine cancer to 7.86-fold in salivary gland carcinoma, and the protein level increases ranged from 1.45-fold in non-Hodgkin’s lymphoma to 8.88-fold in gastric cancer (Figure 3).

### SK1 positivity/high expression rates in cancers

Different cancer types present different rates of SK1 positivity/high expression. We calculated the SK1 positivity/high expression rates in various cancers, and these results are shown in Table 3. The SK1 positivity/high expression rate in estrogen receptor-negative (ER−) breast cancers (58.7%) was higher than that in ER-positive (ER+) breast cancers (27.3%). The average positive rate in breast cancers was 31.1%. The lowest positive rate was found in breast cancer with unknown ER expression status, whereas the highest positive rate (82.2%) was found in tongue squamous cell carcinoma (TSCC; Table 3). Because of the small sample size of the TSCC study (n = 45), selection or other biases would unavoidably affect the result.

**Table 3:** SK1 positive/high expression in variety cancers and non-cancer tissues.

Cancer types	Total	SK1 positive/high expression (%)
ER+ breast cancer	2202	602(27.3)
ER- breast cancer	812	477(58.7)
Breast cancer(unknow)	1287	257(20.0)
Breast cancer(total)	4301	1336(31.1)
Salivary gland carcinoma	159	85(53.5)
Esophageal cancer	136	110(80.9)
Gastric Cancer	412	263(63.8)
Colon cancer	229	170(74.2)
Prostate cancer	227	163(71.8)
Glioma	243	100(41.2)
Neuroblastoma	142	73(51.4)
Thyroid Cancer	42	29(69.0)
HNSCC	18	8(44.4)
TSCC	45	37(82.2)
Non-cancer tissues	750	255(34.0)

**Abbreviations** SGC: salivary gland carcinoma; HNSCC: Head and neck squamous cell carcinoma; TSCC: Tongue squamous cell carcinoma; NA: not available.

### SK1 enzyme activity in cancer tissues

**Figure 4:**
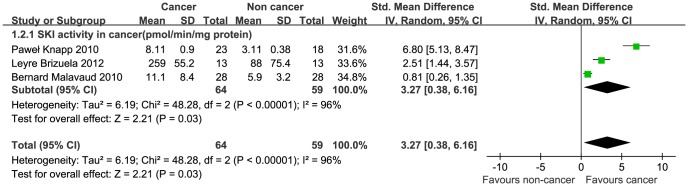
Forest plot of the association between SK1 enzyme activity and cancer tissues. A random-effect model was used. The pooled SMD is symbolized by a solid diamond at the bottom of the forest plot, the width of which represents the 95% CI.

Four studies that measured SK1 enzyme activity were evaluated. One was excluded because the SK1 activity was measured in the blood [43]. A forest plot showed that the SMD (95% CI) were 3.27 (0.38–6.16), with P = 0.03 (Figure 4). One study by Leyre (2012) reported an obvious excess of SK1 enzyme activity levels, compared with the other 2 studies [14]; the heterogeneity was so remarkable (P <0.00001, I^2^ = 96%) that it could not be altered by omitting any single study from the sensitivity analysis; therefore, we cannot draw a firm conclusion regarding a relationship between SK1 activity and cancer.

### Five-year and overall survival rates and SK1 expression in cancer tissues

**Figure 5:**
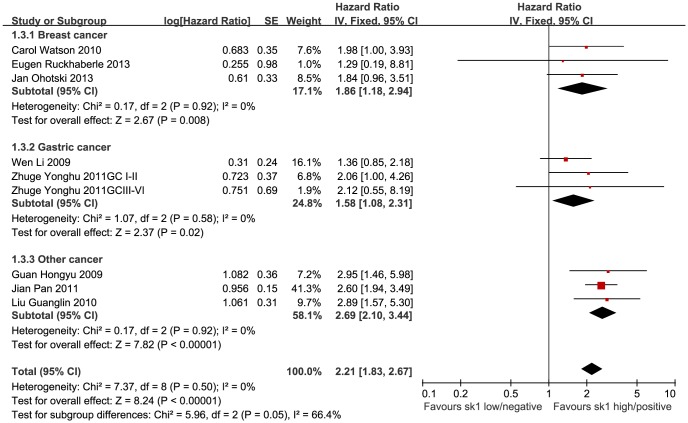
Forest plot of association between SK1 expression and 5-year survival. A subgroup analysis for different cancers and fixed-effect model were used. The squares and horizontal lines correspond to the study-specific HR and 95% CI. The areas of the squares reflect the weights (inverse of the variance). The diamond represents the summary HR and 95% CI.

**Figure 6:**
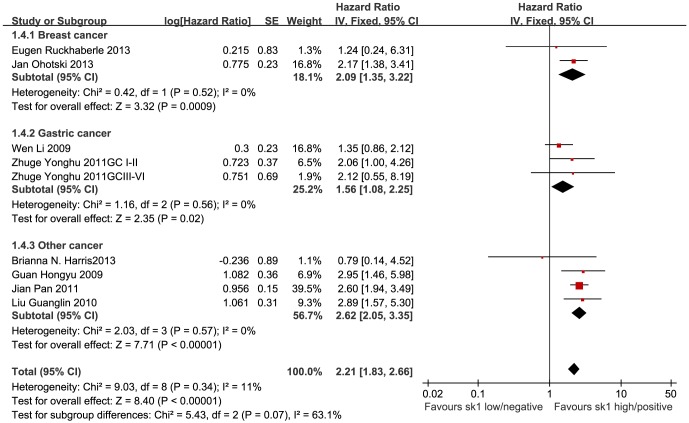
Forest plot of association between SK1 expression and overall survival. A subgroup analysis for different cancers and fixed-effect model were used. The squares and horizontal lines correspond to the study-specific HR and 95% CI. The areas of the squares reflect the weights (inverse of the variance). The diamond represents the summary HR and 95% CI.

The HR values, log[HR], and SE[log(HR)] were either obtained directly from study data or extracted from survival curves, according to the methods described by Parmar or Tierney [20], [21]. Generic inverse variance (IV) and subgroup analyses were adopted according to the types of cancer. As no significant heterogeneity was found, a fixed-effect model was used to perform the meta-analysis. The 5-year survival meta-analysis showed that in cancer patients, the HR of SK1 positivity/high expression was 1.86 (95% CI, 1.18–2.94) for breast cancer (P = 0.008), 1.58 (1.08–2.31) for gastric cancer (P = 0.02), and 2.68 (2.10–3.44) for other cancers (P < 0.0001). The total HR among all the cancers was 2.21 (95% CI, 1.83–2.67; P < 0.00001). SK1 positivity/high expression is thought to associate with a shorter 5-year survival duration in cancer patients (Figure 5). For overall survival, the HR was 2.09 (95% CI, 1.35–3.22) for breast cancer (P = 0.0009), 1.56 (1.08–2.25) for gastric cancer (P = 0.02), and 2.62 (2.05–3.35) for other cancers (P < 0.00001). The total effect HR was 2.21 (95% CI, 1.83–2.66; P <0.00001). These data imply that SK1 positivity/high expression is related to overall survival in various cancers (Figure 6).

### Publication bias

**Figure 7:**
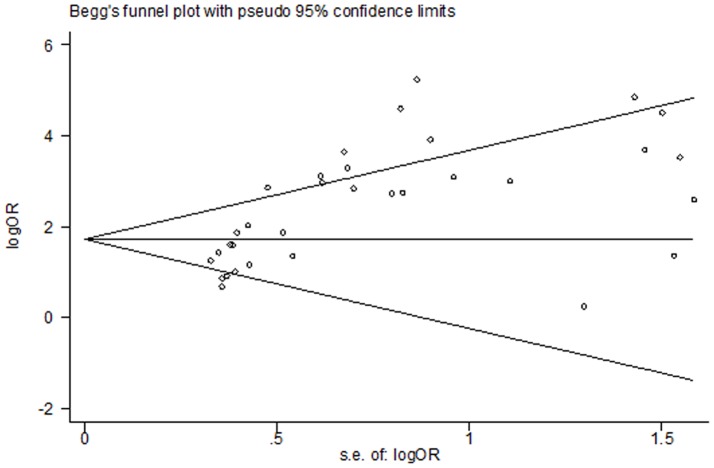
Funnel plot for publication bias in an association analysis of SK1 expression among cancer, adjacent non-cancer, and benign tissues. Begg’s linear regression test was used, and P = 0.059, 0.009, 0.040, and 0.176 in a subgroup analysis of SK1 positivity and high expression in cancer and non-cancer tissues, respectively (Figure 2).

**Figure 8:**
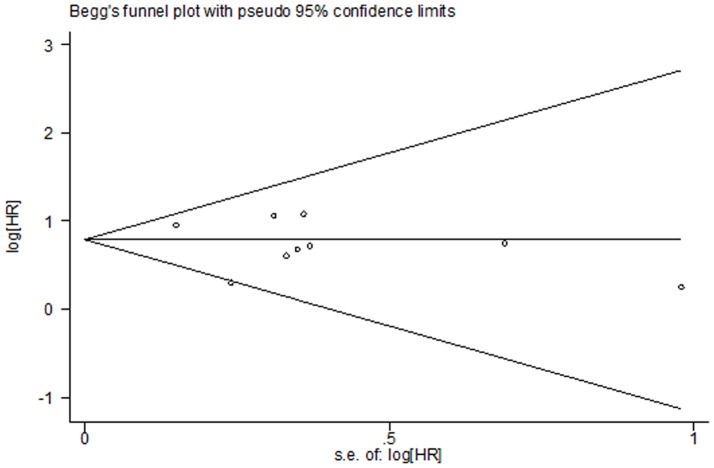
Funnel plot for publication bias regarding 5-year survival. Begg’s linear regression test was used; P = 0.754.

**Figure 9:**
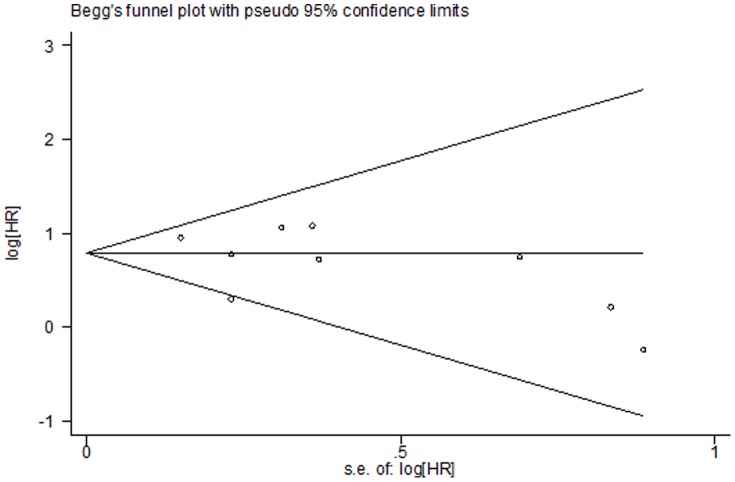
Funnel plot for publication bias regarding overall survival. Begg’s linear regression test was used; P = 0.175.

Egger’s test was performed to assess the publication bias in the literature. In a subgroup analysis of SK1 expression in cancer, adjacent non-cancer, and benign tissues, publication bias was found for cancer versus adjacent non-cancer (P = 0.009) and cancer versus benign tissues (P = 0.04); the funnel plot was not symmetrical. No publication bias was found for cancer versus non-cancer (P = 0.059) and adjacent non-cancer versus benign tissues (P = 0.176; Figure 7). This might be a limitation of our analysis because studies with null findings, especially those with small sample sizes, were less likely to be published. In addition, different test methods were used in these studies. No publication bias was found with regard to 5-year (P = 0.754) and overall survival (P = 0.175; Figures 8 and 9).

## Discussion

The lipid kinase SK1 is expressed extensively in human beings, mice, yeast, and plants and plays an important role in regulating the dynamic balance of the ceramide/S1P rheostat. SK1 overexpression or increased activity leads to S1P accumulation that in turn contributes to prosurvival and anti-apoptotic mechanisms, as well as cancer genesis, metastasis, and drug-resistance. In contrast, ceramide accumulation leads to pro-apoptotic, autophagic, and anti-survival effects. High SK1 expression was detected in many cancers, including those of the lung, breast, ovary, stomach, and kidney.

IHC of formalin-fixed, paraffin-embedded samples is among the most commonly used methods to detect SK1 expression. We evaluated the association of SK1 and different cancers and non-cancer tissues according to the IHC outcomes. In the subgroup and total effect analyses, SK1 positivity/high expression was significantly increased in cancer tissues, compared with non-cancer (adjacent and/or benign) tissues. The OR between cancer and adjacent tissues (2.68) was less than that between cancer and benign tissues (11.94). This finding demonstrates that the SK1 expression level gradually increased in benign, adjacent, and cancer tissues. This might indicate that SK1 expression is associated with degrees of pathological differentiation or heterogeneity in cancer. However, different IHC judgment criteria existed among these studies. Positive and negative were used to judge some, while others were graded according to signs (−, +, ++, and +++). In our meta-analysis, we used negative/low (including − and +) and positive/high (including ++ and +++) to assess SK1 expression in different tissues. That might be partly responsible for the heterogeneity.

When the mRNA and protein expression levels were detected via reverse transcriptase PCR or western blotting, the results were consistent. We found that the SK1 positive/high expression rates varied tremendously between different types of cancer (ranging from 20–82.2%). Differences in SK1 tissue distribution might be the main reason for this variation. Because the sample sizes and detection methods were different, the high heterogeneity prevented us from merging the studies to perform a meta-analysis.

Moreover, only 3 studies [14], [28], [30] reported SK1 enzyme activity, and the interstudy activity level gaps were very wide (Figure 3). In all the 3 studies, the radioassay method was used to detect SK1 activity. This gap might have been introduced by the operator or testing instrument. It might also have been caused by differences in cancer types. To obtain more accurate results, more studies and the evaluation of more samples of the same cancer type with identical detection methods are needed.

Regarding the survival analysis, the follow-up duration in these studies ranged from 30 months to 12 years. We used 5-year and overall survival to evaluate the correlation between SK1 expression and survival in cancer patients. Studies in which the follow-up time was ≥5 years were included in the 5-year survival analysis. Because the overall survival follow-up times differed, we could not acquire concrete data from some of the articles and instead extracted data from survival curves. Therefore, the outcomes might not be very accurate. However, we do not believe that this affected the total outcome.

In our study, SK1 expression was detected in 19 common types of cancer. Some of the more common cancers were the focus of more studies, including breast cancer, which was researched in 7 studies [23], [25], [31], [32], [44], [51], [53], and colon cancer, which was researched in 5 studies[15], [27], [45], [49], [52]. Therefore, different cancers were represented unequally with respect to sample numbers. Meanwhile, the cancer tissue sample and control sample selection methods differed among different studies. In some studies, the cancer and control tissue samples were collected from different areas in the same patient, whereas in other studies, the samples were collected from different patients. Moreover, in these studies, different SK1 detection methods were used and cancer patients of different ethnicities were included. All of these could be sources of heterogeneity. Despite the heterogeneity that was observed in some subgroup analyses, the total outcomes were not affected by the sensitivity analysis.

There were some other limitations and potential biases in our meta-analysis. First, as few (<3) studies regarding disease-specific survival (DSS) and disease-free survival (DFS) were included, we could not perform a meta-analysis of those variables, which are used to evaluate prognosis in cancer patients. Second, this meta-analysis did not discuss the associations between SK1 and ethnicity, tumor size, TNM stage, histological classification, pathological differentiation, lymphatic invasion, and metastasis, all of which are important clinical characteristics in cancer. In addition, only 1 or 2 studies were identified for some types of cancer, and as those sample sizes are insufficient, the associated biases might be remarkable.

In the individual papers, the SK1 expression were found in one or two kinds of cancers in each study. In our meta-analysis, we found that there were relationships between SK1 expression and various cancers, which may imply the possibility of using the SK1 level as the clinical biomarker of cancers.

## Conclusion

Despite some limitations in this meta-analysis, our study still demonstrates that SK1 positivity/high expression is significantly associated with various types of cancers and reduced 5-year and overall survival. Because the SK1 positivity/high rates differ in different types of cancer, it is necessary to further explore whether SK1 might be a predictive biomarker of outcomes in cancer patients.

## Supporting Information

Checklist S1 **PRISMA checklist.**
(DOC)
